# A Computational Analysis of the Function of Three Inhibitory Cell Types in Contextual Visual Processing

**DOI:** 10.3389/fncom.2017.00028

**Published:** 2017-04-25

**Authors:** Jung H. Lee, Christof Koch, Stefan Mihalas

**Affiliations:** Allen Institute for Brain ScienceSeattle, WA, USA

**Keywords:** multiple columnar computational models, mouse primary visual cortex, inhibitory cell types, contextual processing, gain modulation

## Abstract

Most cortical inhibitory cell types exclusively express one of three genes, parvalbumin, somatostatin and 5HT3a. We conjecture that these three inhibitory neuron types possess distinct roles in visual contextual processing based on two observations. First, they have distinctive synaptic sources and targets over different spatial extents and from different areas. Second, the visual responses of cortical neurons are affected not only by local cues, but also by visual context. We use modeling to relate structural information to function in primary visual cortex (V1) of the mouse, and investigate their role in contextual visual processing. Our findings are three-fold. First, the inhibition mediated by parvalbumin positive (PV) cells mediates local processing and could underlie their role in boundary detection. Second, the inhibition mediated by somatostatin-positive (SST) cells facilitates longer range spatial competition among receptive fields. Third, non-specific top-down modulation to interneurons expressing vasoactive intestinal polypeptide (VIP), a subclass of 5HT3a neurons, can selectively enhance V1 responses.

## Introduction

**Figure 1:**
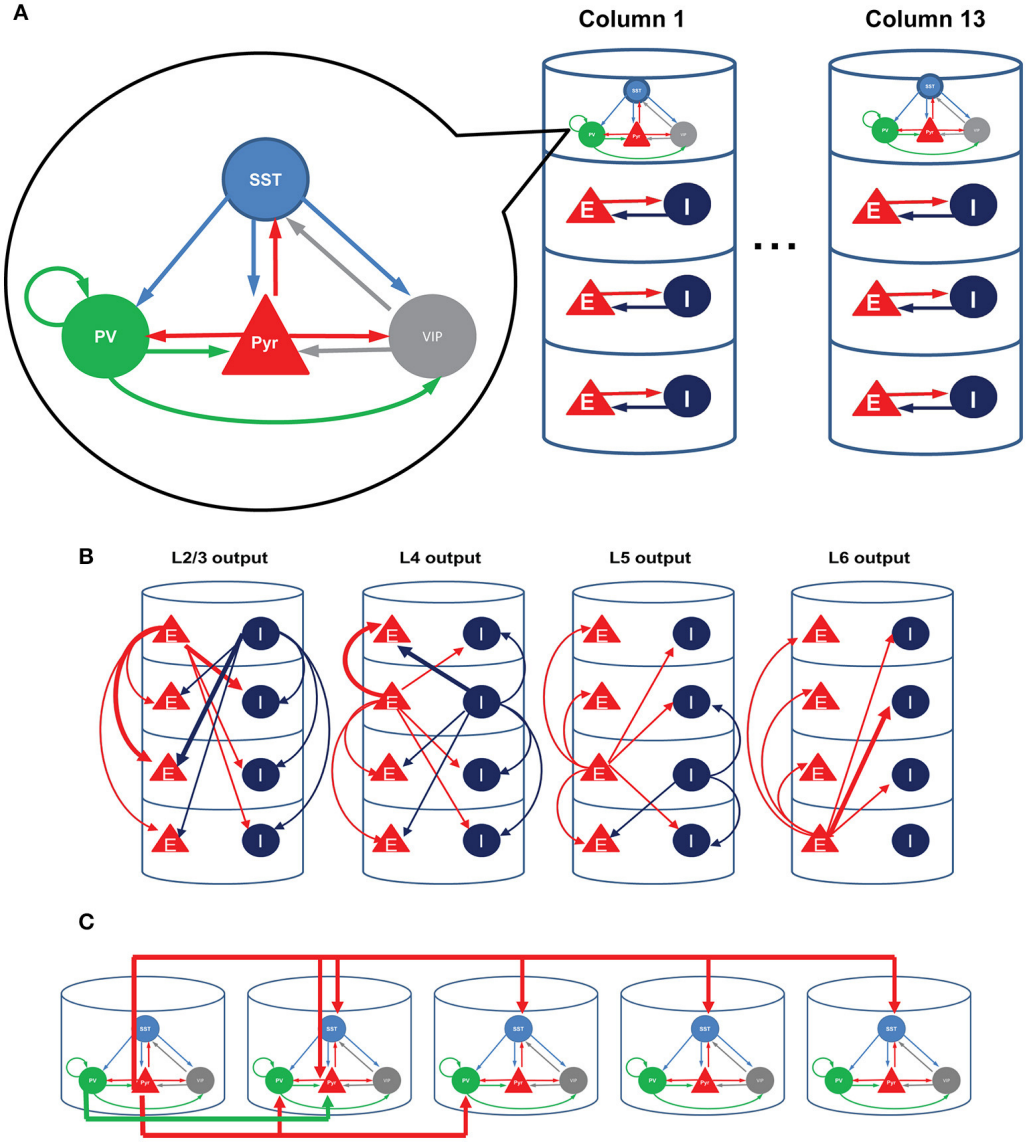
**Structure of the model. (A)** Each single cortical column consists of layer 2/3, layer 4, layer 5, and layer 6. We refined only the superficial layers by incorporating the three inhibitory cell types; all other layers consist of a single excitatory and a single inhibitory cell type, as in the earlier computational model (Wagatsuma et al., 2013). **(B)** The interlaminar connections according to the presynaptic sources. Thick arrows show connections whose probability is higher than 10%. **(C)** The four-types of inter-columnar connections among superficial neurons including short-range PV and long-range SST inhibition; no other intercolumnar connections are considered in this paper.

Inhibitory cells have been considered crucial in regulating neural activity, but a mechanistic understanding of their functional roles remains elusive. In part this is because the inhibitory cell types are multifarious in their morphologies and characteristics. Recent developments of transgenic and optogenetic manipulation demonstrates that the diversity of interneurons can be mapped onto a finite number of classes (Rudy et al., 2011; Kepecs and Fishell, 2014; Tasic et al., 2015). For instance, Rudy et al. (2011) found that nearly all neocortical inhibitory cell types express one of the three genes PV, SST, and 5HT3a exclusively, with roughly 40% of 5HT3a cells expressing VIP. Moreover, PV, SST, and VIP expressing cells have distinctive connectivity (Pfeffer et al., 2013). PV cells inhibit pyramidal cells and themselves, SST cells inhibit all other cell types except themselves, and VIP cells mainly suppress SST cells (Figure 1A).

Recent experiments have corroborated a link between these three inhibitory cell types and distinctive functions. PV cells regulate sensory signal processing in the barrel cortex (Cardin et al., 2009) and modulate the gain of visual neurons (Lee et al., 2012). SST cells participate in the surround suppression (Adesnik et al., 2012). VIP cells thought to be associated with disinhibition of pyramidal cells (Pfeffer et al., 2013; Fu et al., 2014, 2015) are activated during negative feedback (Pi et al., 2013) and mediate top-down modulation to V1 (Zhang et al., 2014).

We here use a computational model of V1 to investigate how the three inhibitory cell types modulate cellular responses in contextual visual signal processing. We focus on studying their roles in regulating interactions among visual neurons with distinctive receptive fields (RFs), for a better knowledge of the interactions among RFs can expose the neural mechanisms underlying contextual spatial processing (Albright and Stoner, 2002). To examine the role of cell type specific connectivity among inhibitory neurons, we used a minimalistic approach in which we started from an existing columnar model (Wagatsuma et al., 2013; Potjans and Diesmann, 2014), and modified only a small number of variables by adding superficial layer circuits incorporating PV, SST and VIP cells. We modeled multiple such nearby columns by assuming that each column responds to a unique RF.

Our simulations demonstrate individual roles for each of the three inhibitory cell types in processing spatial scene context. Firstly, PV cells control the gain of V1 responses and shape the spatial profile of the model response, and could account for the insensitivity of V1 neurons to homogeneous surfaces (Albright and Stoner, 2002). Secondly, SST cells facilitate the competition between objects in the visual scene, thereby effectively enhancing figure-ground contrast. Lastly, a non-specific activation of VIP cells can selectively enhance the responses to preferred stimulus due to coordination among the three inhibitory cell types.

## Results

### Input/output relations for a microcircuit with the three inhibitory cell types

We here consider the superficial layer circuits consisting of pyramidal (Pyr), PV, SST, and a subset of 5HT3a positive neurons (about 40%) that express VIP (Rudy et al., 2011). We adopt the circuit diagram reported by Pfeffer et al. (2013) for the superficial layer 2/3 (Figure 1).

**Figure 2:**
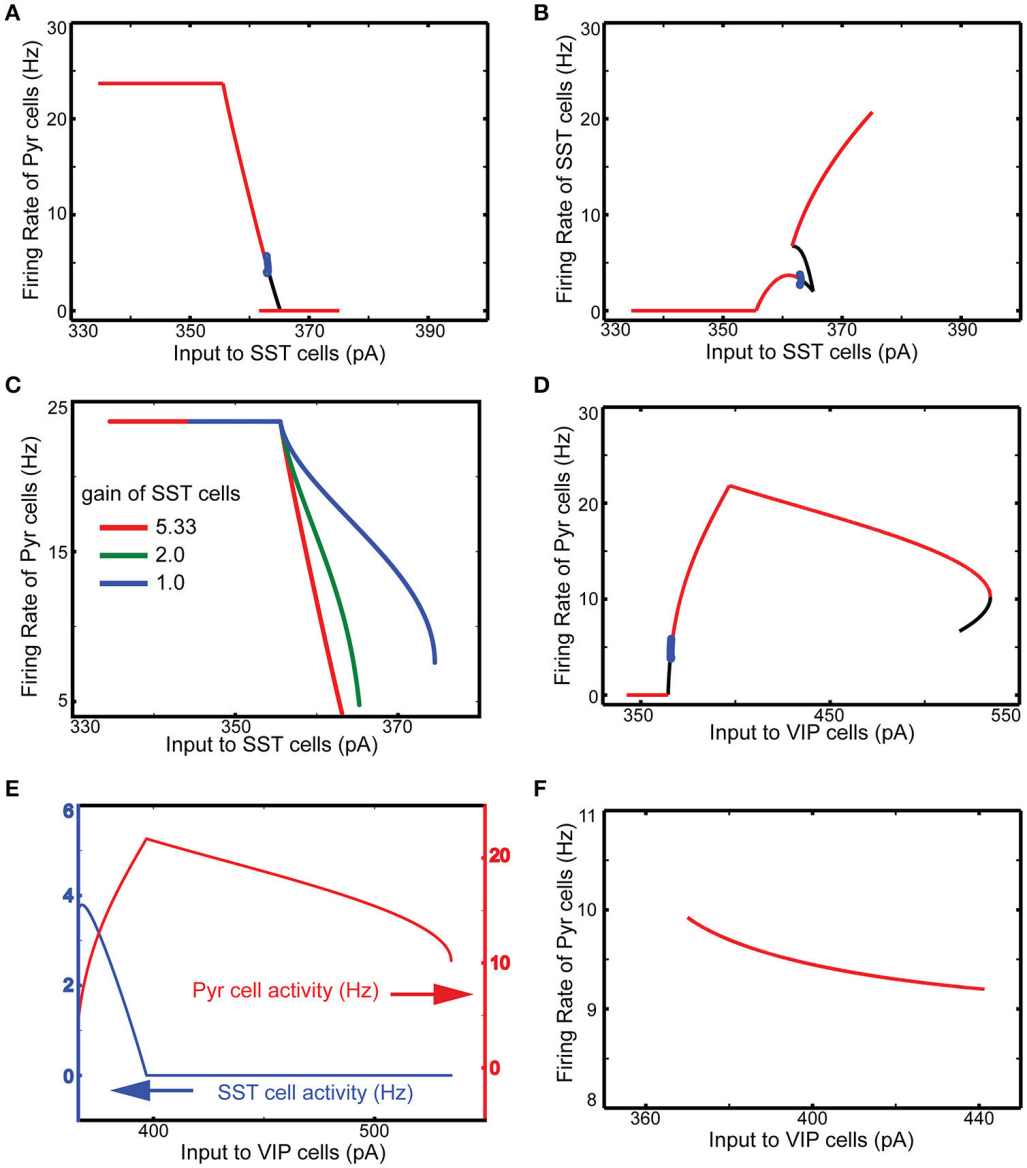
**Qualitative analysis of the functional roles of SST and VIP cells**. The red, black and blue lines represent the stable, unstable steady and periodic solutions of the firing rate equation for the excitatory cells. **(A)** SST input has an overall inhibitory influence onto Pyr activity. **(B)** SST cell activity grows very rapidly. **(C)** The speed of decrease of Pyr cell activity is dependent on the gain of SST cells. **(D)** Importantly, external inputs to VIP cells first lead to a counter-intuitive disinhibition of pyramids before VIP activity shuts down SST firing as well as pyramidal cell activity. **(E)** When SST cells are quiescent, VIP input becomes purely inhibitory; only steady stable solutions are displayed in the panel. **(F)** VIP input cannot disinhibit Pyr cells without VIP-SST connection here set to be zero.

We first perform a qualitative analysis to better understand the interactions among these four cell types in isolation, using a reduced set of four firing rate equations (see Methods). The red, black and blue lines in Figure 2 represent the stable, unstable steady and periodic solutions of the firing rate equation for the population of excitatory cells. They indicate distinctive effects of SST and VIP cell activity on layer 2/3 Pyr cell activity. As expected, SST cells suppress Pyr cell activity (Figure 2A). The steep decrease of Pyr cell activity can be attributed to the rapid increases of SST cell activity (Figure 2B). When the input to SST cells is higher than ~365 pA, SST cell activity is strong enough to silence all other cell types. After this point, SST cells receive no internal interactions but are purely driven by external inputs: the firing rate can be predicted by its neuronal gain function (see Methods). Pyr cell activity reduces more slowly (Figure 2C), when we reduce SST cell activity by decreasing the coefficient of the gain function (see Methods), confirming that the steep decrease of Pyr cell activity results from the rapid increases of SST cell activity.

Contrariwise, increasing the input to VIP cells disinhibits Pyr cells (Figure 2D). As the input to VIP cells increases, SST cell activity decreases (Figure 2E). As a result, Pyr cells receive net reduced inhibition since VIP cells only weakly inhibit Pyr cells. On the other hand, if the external input to VIP is below the threshold (360 pA), there is no inhibition impinging onto SST cells, making SST cell activity strong enough to suppress Pyr cell activity. We also note that SST cells become quiescent at the critical point when Pyr cell activity starts decreasing (Figure 2E). That is, if VIP cell activity continues to grow after this point, its effect on Pyr cells becomes purely inhibitory. Without inhibition from VIP to SST cells, the input to VIP cells suppresses Pyr cells (Figure 2F), confirming that VIP cells can disinhibit Pyr cells by suppressing SST cells. To better understand how these cell types contribute to contextual information processing, we use computational models of V1 and discuss simulation results below.

### Dynamic interaction among superficial layer cells can be critical for the responses of the whole column

We first embed the superficial circuit into the columnar model of Wagatsuma et al. (2013), consisting of 19,294 leaky integrate-and-fire (LIF) units stimulated by barrages of spikes generated with a Poisson process (see Methods for details), which are referred to as external background inputs hereafter. We also use their inter-laminar connectivity scheme (Figure 1B), with the added assumption that all three superficial interneurons are treated the same with regard to inter-laminar connections (see Methods). Supplemental Figure 1 shows the spontaneous activity in all four layers. As seen in the figure, all cell types fire asynchronously, and inhibitory cells fire more strongly than excitatory cells. Superficial layer cells show distinctive responses depending on the excitability of inhibitory cells, which are regulated by the external background inputs. We note that VIP and SST cells appear to be active exclusively. When VIP cells are active, SST cells do not fire (Supplemental Figure 2A). In contrast, when SST cells' excitability is enhanced by the stronger background inputs, VIP cells stop firing (Supplemental Figure 2B). This is because of mutual inhibition between the two cell types; indeed the two cell types were competitive with each other (Karnani et al., 2016). In addition, superficial layer cells can also show synchronous and oscillatory responses when VIP cells' excitability is reduced (Supplemental Figure 2C). The frequency of oscillatory responses appears to be slightly below 20 Hz. It is largely consistent with that of slow oscillation generated by low-threshold spiking interneurons known to express SST (Roopun et al., 2010). While the functions of such low-frequency oscillatory response remain debatable, this is beyond the scope of our study. We select default external background inputs (**Table 2**) so that all cell types could fire asynchronously (Supplemental Figure 1).

**Figure 3:**
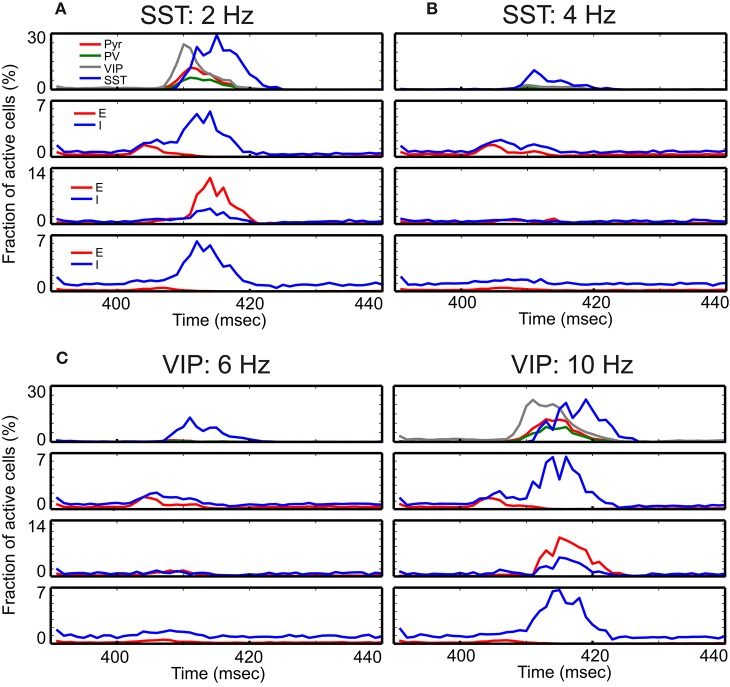
**Time course of cell-type specific cortical activity following transient thalamic inputs**. The thalamic input is modeled as a single 10 msec wave of spikes starting at 400 msec. **(A)** Response of the refined single column with standard parameters responding to the transient thalamic inputs. Thalamic inputs are projected to both excitatory (E) and inhibitory (I) cells in layers 4 and 6. **(B–D)** show the same measure but with different external inputs. The non-default network values chosen are shown at the top of panels. These detailed simulations replicate our qualitative analysis (Figure 2) in which SST activation **(B)** or VIP inactivation **(C)** results in reduced responses, and VIP activation **(D)** results in prolonged responses.

We examine the responses of this refined column to transient thalamic inputs onto layer 4 and layer 6 (Potjans and Diesmann, 2014), averaging cell type activity over 100 independent simulations (Figure 3A). Two observations are germane here. First, excitatory (referred from now on as E) layer 4 cells relay thalamic excitation to other layers. After L4 activation, signals propagate to 2/3 and 5, which is largely consistent with feedforward activation (Schroeder et al., 1998; Lakatos et al., 2010). The onset of inhibitory (referred as I) layer 6 cell activity occurs almost simultaneously with layer 5 E cell activation, resulting from the excitation from layer 2/3 Pyr cells. Second, in the superficial layer, all cell activity is enhanced by thalamic inputs, but the characteristic response times are cell type specific. The onset of VIP cells is the earliest and is followed by Pyr, PV, and SST cell activation; see Discussion. This is not surprising since VIP cells receive weak inhibition unless SST cells are active according to the connectivity (Figure 1A). Such early activation of VIP cells justifies the delayed activation of SST cells because VIP cells inhibit SST cells.

Our qualitative analysis suggests that activation of SST cells can suppress Pyr cell activity. Indeed, the increased inputs to SST cells does reduce Pyr cell activity in the superficial layer (Figure 3B). More importantly, all other layer activity is also reduced, suggesting that layer 2/3 Pyr cells can drive all other layer cells. Similar results occur when inputs to VIP cells are reduced (Figure 3C). Figure 3D shows that Pyr cell activity increases by stimulating VIP cells to fire more strongly, consistent with our qualitative analysis (Figure 2).

### Model column responses are contextual and dependent on intercolumnar connections

**Figure 4:**
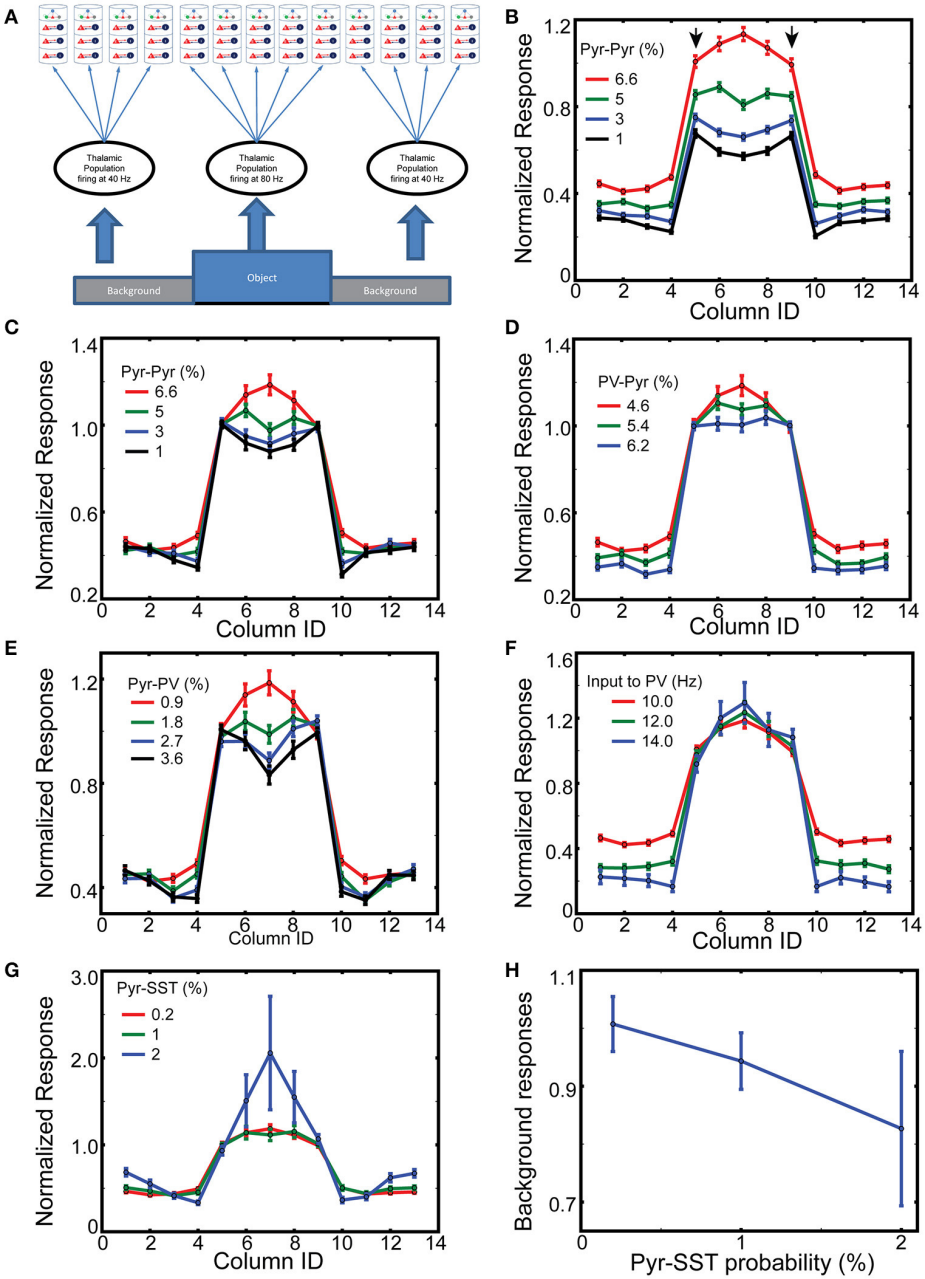
**Spatial context dependent responses of layer 2/3 Pyr cells. (A)** Stimulus layout, with a simplified “object” (corresponding to columns 5–9) superimposed onto a “ground” the nearby columns 1–4 and 10–13; with periodic boundary conditions. **(B)** Normalized responses of Pyr cells between 400 and 500 ms while varying intercolumnar Pyr-Pyr connection probabilities. Error bars represent standard errors. The reference point is the mean value of outputs of edge-columns with default connection probability 6.6% for Pyr-Pyr connections. **(C)** As in **(B)**, except responses are normalized to the mean value of edge responses, on a trial-by-trial basis. Similarly, **(D,E)** display the normalized responses with different connection probabilities for PV-Pyr and Pyr-PV, respectively. **(F)** Column-specific outputs with three different connection external inputs to PV cells. **(G)** Dependency of normalized responses on Pyr-SST connection strength. **(H)** Dependency of background responses on Pyr-SST connection strength.

We next consider a multi-columnar model of V1, combining 13 (instead of 8 as in Wagatsuma et al., 2013) of these columns into an one-dimensional arrangement (Figure 4A).

Since surround suppression, the best studied inter-receptive field interactions within V1, is mediated by long-distance horizontal connections among superficial layers (Adesnik et al., 2012), we analyze how the three layer 2/3 inhibitory cell types contribute to intra- and inter-columnar interactions. We assume that each cortical column is associated with an individual receptive field (RF) and that all columns are connected with one another through superficial-superficial connections only (Figure 1C). We implement two types of di-synaptic inhibitory connections (Figure 1C; di-synaptic because excitation terminates onto interneurons that, in turn, inhibit their postsynaptic local targets): one is a long-range excitatory connection targeting SST cells, and the other a short-range connection targeting PV cells (Adesnik et al., 2012). We also include short-range excitatory Pyr-Pyr and inhibitory PV-Pyr connections among nearest neighbor columns (Figure 1C). In the following, we keep all intra-columnar connections fixed, varying the number of inter-columnar connections and external input strengths to layer 2/3 cell types (see Methods).

Boundary detection is of fundamental importance to visual perception. Most boundary detection schemes identify discontinuities in the image, which can range from discontinuities in luminosity (edges) to discontinuities in higher order statistics (texture boundaries). V1 neurons are generally insensitive to homogenous surfaces (Albright and Stoner, 2002), which can be explained by inhibition from nearby cells with similar responses. To model which inhibitory cell type predominantly contribute to this process, we study how our 13 columns model responds to a simple figure-ground stimulus. Columns 5–9, corresponding to the “figure,” are considered edge- and surface-columns, with the corresponding four neighbor on each side being “ground” (Figure 4A). The corresponding thalamic cells fire at 80 and 40 Hz, respectively, 400–500 ms after onset of simulations. As expected, layer 2/3 pyramids respond prominently within columns corresponding to the “figure” (Supplemental Figure 3), and only weakly in “ground” columns. Following stimulus offset, their response wanes to spontaneous firing.

To compare responses among columns, we normalize the column-specific outputs (firing rates of layer 2/3 Pyr cells) to the mean value of the two edge-column outputs in each simulation during 200 ms after the onset of thalamic inputs. The mean response and standard errors from 100 independent simulations of layer 2/3 Pyr cells are displayed in Figure 4B. The reference value is the mean value of edge column responses marked by arrows when the Pyr-Pyr inter-columnar connection probability is 6.6%. As seen in the figure, the figure-responses are context-dependent, and the exact spatial profile is determined by the intercolumnar interactions. When Pyr-Pyr interaction is strong (red lines), the response to the surface is stronger than that to the edges, whereas edge columns generate stronger outputs (blue and black lines in Figure 4B) when Pyr-Pyr interaction is feeble.

However, this comparison only shows the average level of outputs over 100 trials, and does not necessarily suggest the reliability of the contextual responses generated in each trial. Thus, we normalize the model outputs to the mean responses to the edges in each simulation and display them in Figure 4C to confirm that inter-columnar connections can induce contextual responses on a trial-by trial basis; we use this trial-by-trial basis normalization for the rest of Figure 4 and Supplemental Figure 4. The inhomogeneous responses of model columns, dependent on intercolumnar connections, can be explained by the boundary effects induced by the discontinuity existing at the edges. The edge columns receive less excitation and inhibition through inter-columnar connections than surface columns do, since the background induces less Pyr cell activity in the corresponding columns. That is, edge columns will have less disynaptic inhibitory inputs than surface columns if the net intercolumnar inputs are effectively inhibitory. We test this hypothesis by increasing the connection probability for Pyr-PV and PV-Pyr, both of which enhance intra-columnar inhibition. As expected, the response of the surface columns is reduced when its strength is increased (Figures 4D,E). In addition, the short-range inhibition can reduce surface column responses more effectively when the Pyr-Pyr connection probability is lowered to 1% (Supplemental Figure 4). These results do confirm our hypothesis. However, it is possible that the globally enhanced inhibition from PV to Pyr cells is capable of generating edge dominant responses. To test this possibility, we perform the same simulations but with enhanced background inputs to PV cells; specifically, we increase the frequency of spike trains carried by a single external fiber to PV cells. As seen in Figure 4F, the responses of surface columns are not reduced when the inhibition is globally enhanced, confirming that the intercolumnar inhibition mediated by PV cells is necessary for realizing edge-dominant responses.

Next, we examine if the functional long-range inhibition, mediated by Pyr cells making long-range connections onto SST cells (Figure 1C), can modulate the spatial profile of column responses by increasing the connection probability for Pyr-SST cells. The strengthened Pyr-SST connections enhance the response of surface columns (Figure 4G), which is strikingly different from edge-dominant responses (Figure 4E). Specifically, column 7, which receives the strongest excitation due to inter-columnar excitation, generates twice the response of the edge columns. Once again, the responses in Figure 4G are all relative to the edge column responses in each simulation condition. In fact, the spiking activity of Pyr cells in background columns becomes less as the connections from Pyr to SST cells increases (Figure 4H); this indicates that Pyr-SST cell connections can effectively reduce surround suppression to edge- and surface-columns.

This raises the question: Why does long-range inhibition via SST cells behave differently from short-range inhibition mediated by PV cells? All SST cells in the figure-columns receive inter-columnar excitation from 4 figure and 4 ground columns. That is, there is no spatial gradient of inhibition among figure-columns, suggesting that long-range inhibition is insensitive to the boundary effects inducing edge-dominant responses. In addition, once inter-columnar connections from Pyr to SST cells are increased, PV cell activity decreases due to the inhibition from SST to PV cells. Together, short and long-range inhibition generate paradoxical effects.

### The coordination between SST and VIP cells can selectively suppress neural responses to the backgrounds

**Figure 5:**
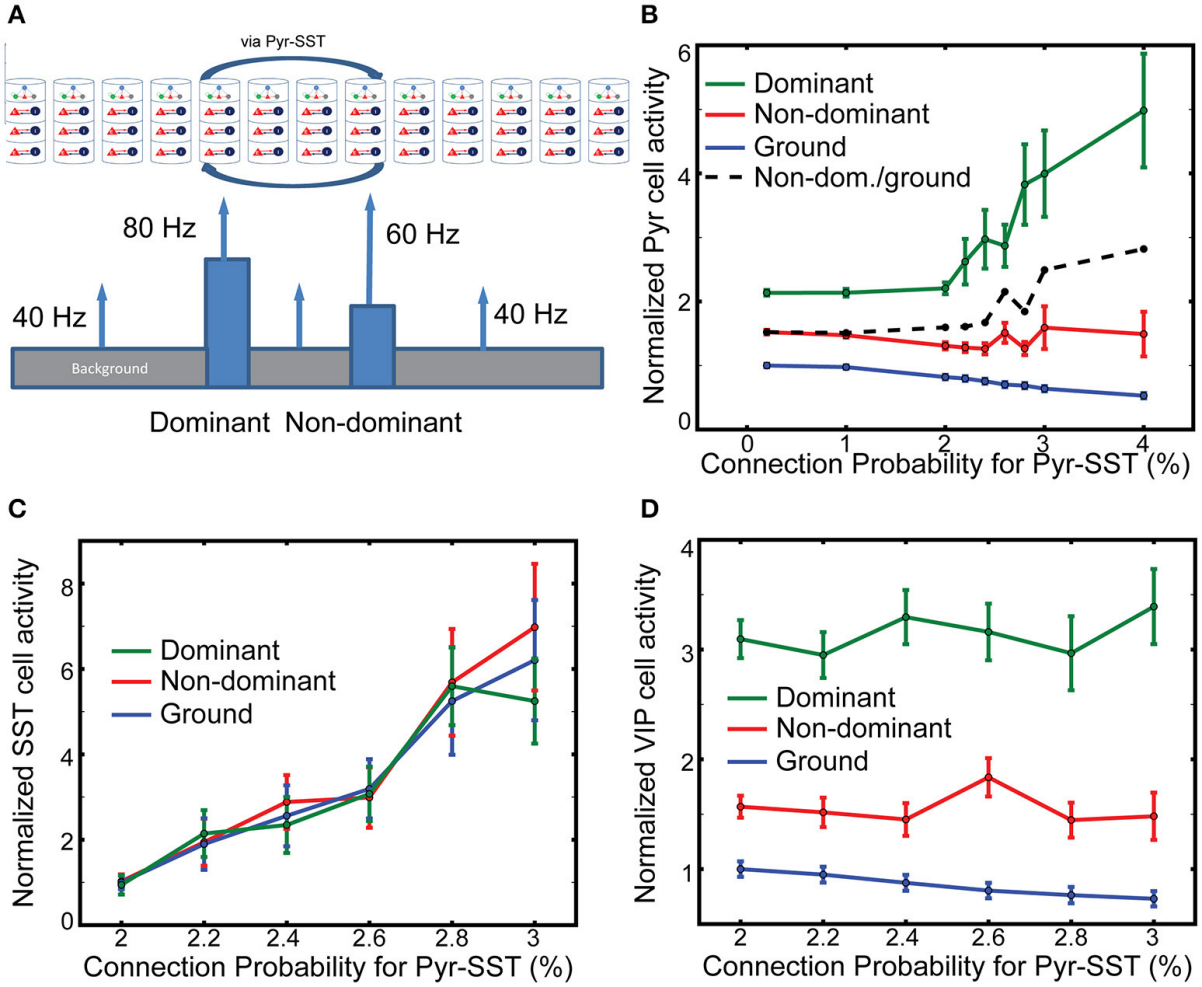
**The effects of long-range inhibition on responses. (A)** Stimulus layout of two, one-columnar wide objects. **(B)** Firing rates of layer 2/3 Pyr cells normalized to ground-evoked responses with the lowest connection probability (0.2%). The ratio of non-dominant to ground responses is shown in the black dash line. **(C,D)** SST and VIP cell activity in all columns, respectively. The reference values in them are the background-evoked responses with the lowest connection probability (2%). Note that the normalized firing rate of SST cells does not vary across the input.

Figure 4G indicates that SST cells can effectively control the competition over large spatial scales between figure and ground. However, a strong competition may be undesirable under certain circumstances. For instance, when two objects are in close proximity, so that the two corresponding columns could inhibit each other via Pyr-SST cell connections, the dominant object could prevent the non-dominant one from evoking a response. Therefore, we examine how long-range inhibition mediated by SST cells modulate our model's responses to multiple objects, here a dominant and a non-dominant object embedded in the background (Figure 5A). The former induces 80 Hz firing in its associated thalamic cells and targets cortical column 5, while the latter triggers 60 Hz thalamic firing, projecting to column 8. All other columns receive thalamic afferents firing at 40 Hz, corresponding to ground. In the following, we designate a column as dominant, non-dominant and ground columns according to their thalamic sources below.

We evaluate the effects of SST-cell mediating long-range inhibition on Pyr cell activity in dominant and non-dominant columns by increasing the connection probability for Pyr-SST cells by a factor of 20. After performing 100 independent simulations with fixing connection probability for Pyr-SST to be 0.2%, we calculate the mean firing rate of Pyr cells for ground and use it as a reference value. The normalized responses to the two stimuli do not change noticeably until Pyr-SST is increased by ten, from 0.2 to 2% (Figure 5B). Increasing inhibition further accentuates the response of the dominant stimulus. This result can be explained by the reduction of surround suppression from background/non-dominant to the dominant columns. We also note that Pyr cell activity in the non-dominant column also increases if it is normalized to the responses to ground (the dashed black line in Figure 5B). This is somewhat surprising since the spiking activity of Pyr cells in the dominant column, which can project di-synaptic inhibition to Pyr cells in the non-dominant column, is greatly enhanced.

To investigate the mechanisms underlying the minimal reduction of non-dominant column responses, we calculate the firing rates of SST cells in the dominant, non-dominant and ground columns. We expect disparate SST cell activities among columns, since SST cells in the columns receive different synaptic inputs. Figure 5C shows the results depending on the connection probability for Pyr-SST cells. All firing rates are normalized to the background-evoked SST cell activity, with the lowest connection probability (2%) for Pyr-SST cells. The disparity in SST cell activity among columns is not high, suggesting that the long-range inhibition suppresses Pyr cells equally across all columns. Such uniform inhibition can most effectively reduce Pyr cell in the ground columns and reduce surround suppression to both dominant and non-dominant columns; this reduced surround suppression to the non-dominant column may compensate for the enhanced di-synaptic inhibition from the dominant column.

It should be noted that VIP cell activity is the highest in the dominant column and the lowest in the ground columns (Figure 5D). In other words, VIP cells can provide stronger inhibition to SST cells in the non-dominant column than in the ground columns, which renders SST cell activity relatively uniform across all columns (Figure 5C).

### VIP cells can work as a gate keeper for the contextual information from higher order cortical areas

Finally, we wish to elucidate the potential mechanisms underlying an elegant experiment that genetically targeted a subset of neurons in cingulate cortex in the mouse (Cg), which directly projects to V1, activating or inactivating these via optogenetic tools (Zhang et al., 2014). This enhanced or reduced orientation-tuning of V1 neurons, possibly via local activation of superficial VIP neurons. Remarkably, Cg activation also enhanced behavioral performance of the mice in an orientation discrimination task (Zhang et al., 2014). The author noted that it is likely that the laser light stimulating the ChR2 expressing neurons activated the entire Cg, leading to the possibility that the observed gain modulation is induced by non-specific top-down signaling. How could non-specific VIP cell activation selectively enhance neural responses to the preferred orientation?

**Figure 6:**
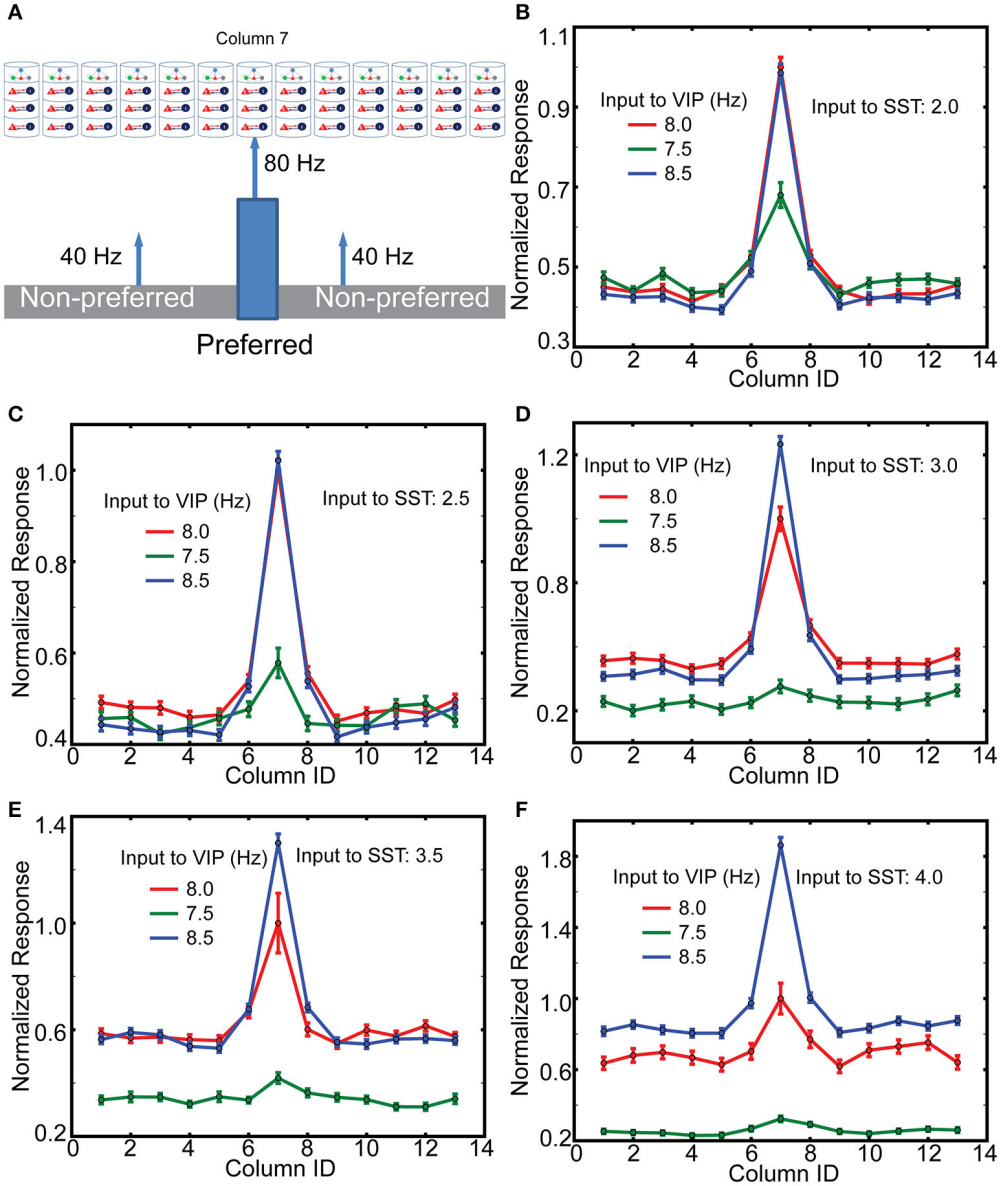
**The effects of top-down inputs to VIP cell onto layer 2/3 pyramids**. **(A)** A single thalamic “object” excites it corresponding cortical column. **(B)** Normalized columnar responses receiving top-down input, in a spatial homogeneous manner, to both VIP and SST cells. Note that, as in previous figures, decreasing VIP activity causes an increase in Pyr cell firing. The reference value is the response of the preferred column with 8 Hz input to VIP cells. **(C–F)** Show the same results but with enhanced levels of input to SST cells.

To gain insight into the mechanisms of top-down gain modulation via VIP cells, we consider V1 responses to an object occupying the RF of a single column. As mouse V1 lacks orientation columns, these simulations do not directly explain the sharpened orientation tuning curve (Zhang et al., 2014). However, we aim to better understand potential mechanisms by which non-specific VIP cell activation selectively enhances V1 responses. In addition, mouse V1 may have “effective” orientation columns (Ko et al., 2013), suggesting that our simulation results with distinct RFs can be a good indicator for gain modulation of orientation tuning curve. For this simulation, we assume that column 7 is preferentially excited (here by lateral geniculate nucleus cells firing at 80 Hz), while all other columns only receive 40 Hz geniculate input (Figure 6A). In addition, cell-type specific external inputs are homogeneous in all columns; this ensures that our model simulates the non-specific top-down signaling. We observe (Figures 6B–F) that layer 2/3 Pyr cell activity in the preferred column is strongly related to the amplitude of synaptic inputs to VIP cells. Furthermore, the responses in non-preferred columns are insensitive to those inputs. We also observe that the effects of non-specific activation of VIP cells are dependent on overall SST cell activity. The gain becomes more pronounced as the inputs to SST cells becomes stronger. These results are consistent with the qualitative analysis (Figure 2E), suggesting that VIP cells disinhibit Pyr cells by suppressing SST cell activity. If SST cell activity is too low, the activation of VIP cells cannot enhance Pyr cell activity but decreases it. It should be noted that Cg indeed activates SST cells as well (Zhang et al., 2014), indicating that the simulations in Figures 6D,E are probably closer to the experimental conditions.

## Discussion

In this study we propose a refined multiple column model of visual cortex which incorporates three inhibitory cell types and cell-type specific connectivity among them. We use these newly developed models to elucidate the functional roles of the three inhibitory cell types in contextual visual signal processing in V1, and our computational analysis indicates cell-type specific functions.

### Inter-columnar interactions play a role in processing contextual information included in visual scene

Depending on the inter-columnar connections, either edge-responding or surface-responding columns can generate dominant responses despite the equivalent level of thalamic inputs (Figure 4). More importantly, PV and SST cells in our models participate in processing spatial contexts in the visual scenes, but their functional roles are distinctive. Short-range inhibition mediated by PV cells generates local spatial gradient of inhibition, allowing V1 neurons to respond to discontinuities induced by the edges of the objects. In contrast, long-range inhibition mediated by SST cells reduces the responses to ground or non-preferred stimuli but increases the responses to preferred stimulus. This selective enhancement, similar to a “winner-take-all” operation, can be explained by the reduction of surround suppression impinging onto V1 neurons responding to the preferred stimulus (Figure 4H). Edge-dominant responses, induced when inter-columnar inhibition is effectively inhibitory, can be useful in detecting the edges of visual objects. This behavior indeed is consistent with the insensitivity of V1 neurons to extended surfaces (Albright and Stoner, 2002). The effective winner-take-all operation introduced by long-range inhibition helps visual neurons distinguish an object from others or from background.

### The three inhibitory cell types coordinate to allow top-down modulation

Our model V1 responses are modulated by the external inputs to VIP cells in a manner consistent with the hypothesis that VIP cells mediate top-down signaling to V1 (Figure 6). Three observations of our simulation results are noteworthy. First, the spiking activity of layer 2/3 Pyr cells responding to the preferred stimulus is selectively enhanced by non-selective activation of VIP cells. That is, even when top-down signaling affects a large portion of V1, the induced effects are pronounced only in the target area, suggesting that top-down signaling need not be strictly target-specific. The same stimulus specificity without corresponding spatial specificity of the afferent input also applies to neuromodulatory input such as acetylcholine. We also note that the lateral inhibition mediated by PV and SST cells can control responses in columns responding to non-preferred stimuli (Supplemental Figures 5A,B).

Second, the gain control mediated by VIP cells is dependent on the overall SST cell activity (Figure 6). The more active SST cells are, the higher the effective gain. Interestingly, both feeble and prominent effects induced by the activation of VIP cells were reported. Lee et al. (2012) found mild changes in the firing rates when VIP cells were directly activated. In contrast, Zhang et al. (2014) found a multiplicative gain modulation when Cg, which innervates VIP cells in V1, was activated. The dependency of the gain on SST cell activity in our model suggests that the difference between the two experiments could be attributed to the fact that SST cell also receives EPSCs from Cg. Thus, Cg activation corresponds to the simulation results with the higher inputs to SST cells. In contrast, when animals passively watch visual stimuli as in Lee et al. (2012), Cg may not be active, and thus SST cell activity could be too low for the disinhibitory control induced by VIP cells.

Third, the enhanced external inputs to PV cells globally reduce Pyr cell activity (Supplemental Figure 5). This is consistent with the subtractive effects experimentally observed (Lee et al., 2012), which can sharpen the tuning curve; (see Lee et al., 2014) for recent discussion. Again, it should be noted that Cg activation induces EPSCs in PV cells as well, suggesting that Cg may activate the PV cells to suppress the responses of Pyr cells responding to the non-preferred stimuli.

In brief, our model proposes that Cg excites all three inhibitory cell types to promote the coordination among the three cell types, allowing non-specific top-down signaling to have target-specificity, which is consistent with a recent experimental/theoretical study suggesting that activation of multiple inhibitory cell types in mouse primary auditory cortex was necessary for generating the context-dependent behavior (Kuchibhotla et al., 2016).

### Limits of our model

We limit ourselves in this study to solely refining the superficial layer for three reasons. First, surround suppression is mainly mediated by horizontal connections targeting superficial SST cells (Adesnik et al., 2012). Second, top-down signals from Cg are known to innervate VIP cells most strongly (Zhang et al., 2014), commonly found in superficial layers (Rudy et al., 2011). Third, the innervating cholinergic axons in superficial layers of V1 induce attentional effects (Chen et al., 2015). In doing so, we ignore cell types in other layers and assume that connections targeting superficial layer inhibitory cells are not cell type specific. Below we discuss the potential drawbacks of these assumptions.

First, we assume that the three inhibitory cell types in layer 2/3 receive equivalent synaptic inputs from other layers. That is, VIP and SST cells in the model may receive overly strong thalamic inputs via layer 4 E cells since these inhibitory cell types receive less ascending excitation from layer 4 E cells. Indeed, this may underlie the discrepancy in the latency of VIP cells between our simulation results (Figure 3A) and experimental observations suggesting that VIP cells' responses are delayed more than PV cells' responses (Mesik et al., 2015). We did not further consider this discrepancy because that VIP cells have only weak connections onto to Pyr and PV, and influence their activity primarily by inhibiting SST cells. Interestingly, the short-latency of VIP cells allows model SST cells to have longer latency than PV cells, consistent with the experimental finding (Ma et al., 2010). As SST cells have stronger impact on Pyr cells, we choose to make SST cells' response characteristic precise.

Second, our simulation results indicate that superficial layer interactions play critical roles in contextual modulation of V1 neuron responses. Of course, this does not exclude the possibility that other layers could participate in mediating lateral interactions. Indeed, a recent survey paper (Jiang et al., 2015) suggested that two cell types, layer 5 horizontally elongated cells (HEC) and layer 1 elongated neurogliaform cells (eNGC) have long horizontal arbors, indicating that they can also mediate the lateral interactions. Furthermore, the interlaminar interactions may also contribute to the lateral interactions. Layer 5 HEC cells receive strong inhibition from layer 2/3 neurogliaform cells (Jiang et al., 2015). That is, surround suppression in layer 5 may also be regulated by superficial layer cells. According to the observed connectivity (Jiang et al., 2015), activation of layer 2/3 Martinotti cells (MC) known to express SST can lead to disinhibition of layer 5 HEC cells mediating surround suppression by inhibiting layer 2/3 neurogliaform cells, making surround suppression in layer 5 increase. This interlaminar interactions may account for the experimental finding that activation of SST cells increased surround suppression in layer 5 (Nienborg et al., 2013). We also note that layer 5 MC may contribute to the surround suppression in layer 4 via their vertical axons (Xiang et al., 1998; Jiang et al., 2015), which can underlie the orientation-tuned suppression in layer 4 (Self et al., 2014).

Third, we ignore many known complications. Examples of these include cell type-dependent mechanisms such as firing rate adaptation (Kawaguchi, 1997), multiple inhibitory cell types in other layers (Markram et al., 2004), dynamic synapses with short- and long-term plasticity (Beierlein et al., 2003) and more sophisticated thalamic input (Jones, 2001).

We feel that at this point in the exploration of cortex, it is best to proceed step-by-step in a systematic manner rather than generating ill-understood biophysical models with very large degrees of freedom. The impact of all such simplifications will be addressed in future studies. Currently, our institute is collecting necessary data to incorporate them into the next generation computational models of V1, and we will probe the updated models to study neural correlates underlying contextual visual information. Also we plan to incorporate cell-type specific cellular mechanisms to further study the functional roles of inhibitory cell types by using generalized LIF neuron model capable of reproducing *in vitro* physiological data.

### Comparison to other models

Although inhibitory cell types are very diverse, only a few models considered multiple inhibitory cell types. Traditionally, low-threshold spiking (LTS) and fast-spiking (FS) interneurons have been identified (Kawaguchi, 1997; Kawaguchi and Kubota, 1997), and they have indeed distinct functions (Gibson et al., 1999; Beierlein et al., 2003). This motivated network models with LTS and FS cells. Hayut et al. (2011) studied interactions among Pyr, FS, and LTS cells using firing rate equations. These two inhibitory cell types were also incorporated into the single column consisting of biophysically detailed neurons to study the underlying mechanisms of cortical rhythms (Traub et al., 2005), and a more recent modeling study (Roopun et al., 2010) suggested that LTS cells are associated with deep layer beta rhythms, inspiring more abstract models focusing on the two inhibitory cell types' contribution to interlaminar interactions (Kramer et al., 2008; Lee et al., 2013, 2015).

Earlier studies also investigated the functions of three inhibitory cell types in working memory (Wang et al., 2004), multisensory integration (Yang et al., 2016) and visual signal processing (Krishnamurthy et al., 2015; Litwin-Kumar et al., 2016). The last two focused on functions of inhibitory cell types in shaping orientation tuning of V1 neurons. Litwin-Kumar and Doiron (2014) studied underlying mechanisms of subtractive and divisive normalization, and Krishnamurthy et al. (2015) investigated how long-range connections targeting SST cells contribute to surround suppression. Our approach is distinct from these two studies in three ways. First, we studied superficial layer interactions in the context of other layers, some of which directly interact with LGN; both studies modeled superficial layer only. Second, we also considered both long-range and short-range di-synaptic inhibition among receptive fields. Third, we estimated V1 response to more general visual objects, rather than orientation tuning curve.

## Methods

Our model is based on the multiple column model proposed by Wagatsuma et al. (2013). In the original model, the eight columns interact with one another via excitatory synaptic connections between superficial layers. Those intercolumnar connections target excitatory and inhibitory cells. Excitatory-excitatory connections reach the nearest columns only, whereas excitatory-inhibitory connections reach all other columns. Here we modified this original model by incorporating the three inhibitory cell types in superficial layers and their cell-type specific connectivity within and across columns to study functional roles of each type in interactions across columns.

We used the peer-reviewed simulation platform “NEST” (Gewaltig and Diesmann, 2007) to build a refined model. All cells in our model are identical “leaky-integrate-and-fire” (LIF) neurons whose postsynaptic currents decay exponentially, and we used NEST-native neuron models. Specifically, we modeled superficial layer cells and other layer cells using “iaf_psc_exp_multisynapse” and “iaf_psc_exp” neuron models, respectively. These two neuron models are identical in terms of internal dynamics for integration and spiking, but the former allows multiple synaptic ports, each of which can have distinctive postsynaptic dynamics. The multiple postsynaptic dynamics are necessary for neuron models to integrate synaptic inputs from multiple types of presynaptic sources. Table 1 shows the parameters for neurons and synapses used in our model.

**Table 1:** **Parameters for the network**.

**Neuron parameters**		**Decay time constants (ms)**	
τ_*m*_	10 ms	Pyr→Pyr	2.0
*V*_*th*_	−50 mV	PV→Pyr	6.0
*V*_*reset*_	−65 mV	SST→Pyr	7.5
τ_*ref*_	3 ms	VIP→Pyr	6.2
*C*	250 pF	Pyr→PV	2.0
**Peak currents (pA), *w* ±δ *w***		PV→PV	4.3
Default excitatory	175.6 ± 17.6	SST→PV	3.4
Default inhibitory	−702.4 ± 70.2	Pyr→SST	2.0
PV→Pyr	−466.7 ± 46.7	VIP→SST	10.4
PV→PV	−638.1 ± 63.8	Pyr→VIP	2.0
PV→VIP	−140.04 ± 14.0	PV→VIP	4.3
SST→Pyr	−200.0 ± 20.0	SST→VIP	3.4
SST→PV	−228.6 ± 22.9	Default exc	0.5
SST→VIP	−525.8 ± 52.6	Default inh	0.5
VIP→Pyr	−76.2 ± 7.62		
VIP→SST	−66.7 ± 6.7		
L4E→Pyr	245.84 ± 24.6		
**Synaptic delay (msec), *d* ±δ *d***		**Neuron #**	
Intracolumnar exc.	1.5 ± 0.75	L2/3	E:5171 I:1459
Intracolumnar inh.	0.75 ± 0.375	L4	E:5479 I:1370
Intercolumnar exc.	7.5 ± 3.75	L5	E:1213 I:266
Intercolumnar inh.	3.75 ± 1.88	L6	E:3599 I:737
**Connection probabilities for intercolumnar connections (%)**		**Weighting factors for cell-type specific connections**	
Pyr-SST	0.2	PV-Pyr:SST-Pyr:VIP-Pyr = 1:1:0.125	
Pyr-PV	0.9		
PV-Pyr	4.6	PV-PV:SST-PV:VIP-SST:SST-VIP:PV-VIP = 1:0.857:0.625:1:1	
Pyr-Pyr	6.6		

*The table above lists the parameters regarding the structure of the model used during simulations. The same constants are maintained for all simulations unless stated otherwise. In cell-type specific connections in superficial layers, both presynaptic and postsynaptic cells are one of Pyr, PV, SST, and VIP; presynaptic on the left side of the arrow, postsynaptic on the right. We also strengthened excitatory connections from layer (L) 4 E to Pyr to balance the excitation from L4 E and Pyr impinging onto Pyr, as in Potjans and Diesmann (2014). For all other connections, we used the same parameters used in Wagatsuma et al. (2013)*.

Each cell type in superficial layers of our model has specific presynaptic sources and postsynaptic targets, as reported in Pfeffer et al. (2013). To incorporate the reported cell-type specific connectivity, we estimated cell-type specific population size, connectivity and postsynaptic dynamics in superficial layers. All other layers are equivalent to those in Wagatsuma et al. (2013) with one exception; see Table 1. Below we illustrate the details about our estimates in superficial layers.

### Firing rate model

We used Wilson-Cowan type firing rate equations to have qualitative understanding of dynamics among the four cell types (Pyr, PV, SST, and VIP) in superficial layers. As in our computational model, we did not consider intrinsic properties of each cell type. Instead, all cell types have distinctive connectivity. Multiple non-linear functions have been used as gain functions in firing rate models (Ermentrout and David, 2010). Among them, the square-root curve can approximate the firing rate of a neuron (Ermentrout and David, 2010); for instance, this function can describe the F-I curve of quadratic integrate and fire neurons (Brunel and Latham, 2003). Thus, we select the square-root function as the gain function and determine the parameters (slope and spike threshold) by fitting it to the F-I curve of the leaky integrate and fire neuron used in a computational model; the fitting result is discussed elsewhere (Lee and Mihalas, 2017). Thus, the firing rates of the four cell types can be described by Equation (1):

(1) $$\begin{array}{l} \tau_{m}\frac{df_{e}}{dt}=-f_{e}+5.33\sqrt{\left(I_{e}+S_{ee}f_{e}-S_{ep}f_{p}-S_{es}f_{s}-S_{ev}f_{v}-\theta \right)}\\ \;H\left(I_{e}+S_{ee}f_{e}-S_{ep}f_{p}-S_{es}f_{s}-S_{ev}f_{v}-\theta \right)\\ \tau_{m}\frac{df_{p}}{dt}=-f_{p}+5.33\sqrt{\left(I_{p}+S_{pe}f_{e}-S_{pp}f_{p}-S_{ps}f_{s}-\theta \right)}\\ \;H\left(I_{e}+S_{ee}f_{e}-S_{ep}f_{p}-S_{es}f_{s}-\theta \right)\\ \tau_{m}\frac{df_{s}}{dt}=-f_{s}+5.33\sqrt{\left(I_{s}+S_{se}f_{e}-S_{ev}S_{ev}-\theta \right)}\\ \;H\left(I_{s}+S_{se}f_{e}-S_{ev}S_{ev}-\theta \right)\\ \tau_{m}\frac{df_{v}}{dt}=-f_{v}+5.33\sqrt{\left(I_{v}+S_{ve}f_{e}-S_{vp}f_{p}-S_{vs}f_{s}-\theta \right)}\\ \;H\left(I_{v}+S_{ve}f_{e}-S_{vp}f_{p}-S_{vs}f_{s}-\theta \right) \end{array}$$

where *Ix, fx, Sxy*, θ = 360 pA are the applied current, firing rate, synaptic weight from presynaptic cell *y* to postsynaptic cell *x* and spiking threshold, respectively; where H is the Heaviside step function; where *e, p, s*, and *v* represent Pyr, PV, SST, and VIP cells, respectively. To estimate the weight *Sxy*, we calculated the total synaptic currents during τ_*m*_ = 10 msec using the same parameters used in computational models (see Table 1). Specifically, we set *S*_*ee*_ = 1.98, *S*_*ep*_ = 5.68, *S*_*es*_ = 3.05, *S*_*ev*_ = 0.12, *S*_*pe*_ = 0.55, *S*_*pp*_ = 2.28, *S*_*ps*_ = 0.55, *S*_*se*_ = 0.55, *S*_*sv*_ = 0.36, *S*_*ve*_ = 0.55, *S*_*vp*_ = 0.50, *S*_*vs*_ = 1.48, *I*_*e*_ = 366, *I*_*p*_ = 362, *I*_*v*_ = 370, *I*_*s*_ = 361.

These equations can be considered Wilson-Cowan equation without the correction terms referring to the neurons' inability to fire during their refractory period. We ignored the correction terms since they will be small unless the neurons' firing rates are high. We numerically solved these equations and performed continuation analysis using the open-source numerical analysis package XPPAUT (Ermentrout, 2007).

### Population size

We split superficial layer inhibitory cells into three populations according to Rudy et al. (2011). First, we set 24% of inhibitory cells in an individual column in Wagatsuma et al. (2013) to be vasoactive intestinal peptide-positive (VIP) cells, since Rudy et al. (2011) suggested that 60% interneurons in superficial layers express 5HT3a and 40% of them also express VIP. Second, we set 30% cells to be somatostatin (SST) cells using the average faction found in all layers. Third, we set the rest to be parvalbumin (PV) cells, which are the most common inhibitory cells in the cortex.

Pfeffer et al. (2013) estimated that PV, SST, VIP and unknown types constitute 36, 30, 17, and 16% of inhibitory neurons, respectively. If we include 16% unknown types in the PV cell group, our estimates are consistent with their findings. Especially, the difference in VIP cell fraction can be attributed to the fact that Pfeffer et al. (2013) measured these values in layer 5 as well, in which VIP cells are less common than those in layer 2/3; thus, their estimates of VIP cell fraction will be lower than those in layer 2/3 alone.

### Connectivity among cell types in superficial layers

Wagatsuma et al. (2013) connected excitatory and inhibitory cells in superficial layers by specifying 4 connection probabilities *P*_*EE*_, *P*_*EI*_, *P*_*IE*_, and *P*_*II*_. We used *P*_*EE*_ and *P*_*EI*_ for recurrent connections among pyramidal cells and excitatory projections impinging onto the three inhibitory cell types. That is, the three inhibitory cell populations are equally connected to the pyramidal cell population; the number of synaptic connections between populations is dependent on the size of postsynaptic cell population, as suggested in Potjans and Diesmann (2014).

We connected the three inhibitory cell types to pyramidal cells using the cell-type specific individual neuronal contribution (INC) on inhibition in mouse visual cortex (Pfeffer et al., 2013). Specifically, we first computed the total number of synapses from inhibitory cells to pyramidal cells used in Wagatsuma et al. (2013). Then we split them into three populations using the connection probabilities reported in Pfeffer et al. (2013) as weighting factors; see Table 1. In the same way, we implemented recurrent connections among the three inhibitory cells. Figure 1 illustrates the cell-type specific inhibitory connections used in our model. We note that this connectivity is adopted from the circuit diagram proposed by Pfeffer et al. (2013). Here we added two more connections VIP-Pyr and PV-VIP into them. VIP-Pyr connection was added to capture the functional roles of inhibition projected from VIP cells to pyramidal cells, and PV-VIP cell connection was added due to the reported high INC value, as detailed in the next subsection.

### Postsynaptic currents in superficial layers

We also approximated cell-type specific postsynaptic currents by estimating peak currents and decay time constants from data reported in Pfeffer et al. (2013). These two factors are sufficient to determine the exact shape of postsynaptic currents in LIF neurons if postsynaptic currents decay exponentially. To estimate them for seven cell-pairs (PV-Pyr, SST-Pyr, VIP-Pyr, PV-PV, SST-PV, SST-VIP, and VIP-SST), we measured the heights of row traces of inhibitory postsynaptic charges (IPSQ) given in Pfeffer et al. (2013) and converted them into peak currents using the reference bar. Table 1 displays our estimates. Once we obtained the peak currents, we calculated the decay time constants by dividing IPSQ values with them due to the property of the exponential-decay curve.

For SST-VIP and PV-VIP connections, the given information is not sufficient to specify both peak currents and decay time constants. We estimated the peak currents using the INC. Specifically, we compared INC values between SST-VIP and SST-PV pairs and between PV-VIP and PV-PV pairs. According to the definition in Pfeffer et al. (2013), INC values are the products of IPSQs and connection probability Pcon. As a result, one condition exists: the three unknown variables given below:

(2) $$\begin{array}{r} \frac{{INC}_{SST-VIP,\;PV-VIP}}{{INC}_{SST-PV,\;PV-PV}}=\frac{{P_{con}\times IPSQ}_{SST-VIP,\;PV-VIP}}{P_{con}\times {IPSQ}_{SST-PV,\;PV-PV}}=\frac{{P_{con}\;\times \;Peak}_{SST-VIP,\;PV-VIP}\;{\times \tau }_{SST-VIP,\;PV-VIP}}{P_{con}\;\times {Peak}_{SST-PV,\;PV-PV}\;{\times \tau }_{SST-PV,\;PV-PV}}. \end{array}$$

We first assumed that the two pairs of connections originating from the same presynaptic cells have the same decay time constant to remove one unknown variable. Then, one constraint should be satisfied by the multiplication of connection probabilities *P*_*con*_ and peak currents. Since we used connection probabilities reported in Pfeffer et al. (2013) as weight factors to distribute synaptic connections, the impacts of connection probabilities should be minimal. Thus, we chose *P*_*con*_ = 1 for both SST-VIP and PV-VIP connections and adjusted peak currents properly, which are also given in Table 1. We noted that those values are roughly consistent with the ratio of raw IPSQ peaks provided in Pfeffer et al. (2013).

The estimated decay times (see Table 1) are much longer than 0.5 msec used in Wagatsuma et al. (2013) and also Potjans and Diesmann (2014). Consequently, the pyramidal cells in superficial layers receive enhanced inhibition. To compensate this, we also lengthened the decay time of excitatory connection in superficial layers to 2 ms (Hayut et al., 2011) by keeping the peak currents of excitatory connections at the same level as in Wagatsuma et al. (2013).

### Interlaminar and intercolumnar connections

Superficial layer cells can also interact with other layer cells. For such interlaminar interactions, we ignored individual types of inhibitory cells in superficial layers. That is, all three inhibitory cells types in superficial layer cells are treated equally by other layer cells, and they equally project inhibition to all other layer targets; we used the same connection probabilities specified in Wagatsuma et al. (2013) to connect cell populations across laminar layers.

Although synaptic connections across columns are poorly understood, a line of studies (Adesnik and Scanziani, 2010; Muir et al., 2011; Adesnik et al., 2012; Martin et al., 2014) suggests that superficial layers are connected with one another, via intercolumnar connections, which is consistent with the model proposed by Wagatsuma et al. (2013). Thus, we implemented intercolumnar connections between superficial layers only. As in Wagatsuma et al. (2013), the targets of intercolumnar connections are pyramidal cells in the nearest neighbors and inhibitory cells in both neighbors and distant columns. Throughout this study, periodic boundary condition was used for intercolumnar connections to ensure that all columns receive equivalent synaptic inputs except selective thalamic signals. All intercolumnar connections are identical to the synaptic connections among superficial layer cells within a column except the conduction delay. Since intercolumnar connections are longer than intracolumnar connections, we introduced 5 times longer conduction delays to intercolumnar connections (Table 1).

### External background inputs and thalamic inputs

All cells in our model receive cell type specific background inputs. Following the protocol used in Potjans and Diesmann (Wagatsuma et al., 2013), each cell type receives them via a fixed number of external fibers, each of which carries independent Poisson spike trains; see Table 2 for exact parameters used in our simulations. Each thalamic cell in our model projects independent Poisson spike train at the fixed rate *P*_*r*_ to its targets in layer 4 and 6, randomly chosen according to the connection probabilities adopted from Wagatsuma et al. (2013). As in Potjans and Diesmann (Wagatsuma et al., 2013), we connected 902 thalamic cells to a single column. The three thalamic populations were used, and all thalamic cells start firing 400 ms after the onset of simulations, and their firing lasts 100 msec unless stated otherwise.

**Table 2:** **External background inputs**.

**NUMBER OF EXTERNAL FIBERS**	
**L2/3**	**Pyr:1,600 Inh:1,500**
**L4**	**Exc:2,100 Inh:1,900**
**L5**	**Exc:2,000 Inh:1,900**
**L6**	**Exc:2,900 Inh:2,100**
**BACKGROUND SPIKE RATE (Hz) PER EXTERNAL FIBER**	
**L2/3**	**Pyr:8.0 PV:10.0 SST:2.0 VIP:8.0**
**All other layers**	**Exc:8.0 Inh:8.0**
**PEAK CURRENTS *w* ±δ *w***	
87.9 ± 8.8 pA	

*In our simulations, all cells receive background external inputs via external fibers, each of which carries Poisson spike trains. The cell-type specific fiber number and the frequency of Poisson inputs are given above. We adopted the peak currents from Potjans and Diesmann (2014)*.

## Author contributions

JL, CK, and SM designed research and wrote the paper. JL performed research. JL and SM analyzed data.

### Conflict of interest statement

The authors declare that the research was conducted in the absence of any commercial or financial relationships that could be construed as a potential conflict of interest.
