# Supplementary material for: A Computational Analysis of the Function of Three Inhibitory Cell Types in Contextual Visual Processing
Source: Front Comput Neurosci. 2017 Apr 25;11:28. doi: 10.3389/fncom.2017.00028 (PMC5403882; doi:10.3389/fncom.2017.00028)
Supplement: Supplementary file 1 [file DataSheet1.docx]

Supplementary Material

A Computational Analysis of the Function of Three Inhibitory Cell Types in Contextual Visual Processing

Jung H. Lee^1^*, Christof Koch^1^ and Stefan Mihalas^1^*

*** Correspondence:** Jung H. Lee: [jungl@alleninstitute.org](mailto:jungl@alleninstitute.org), Stefan Mihalas: stefanm@alleninstitute.org

# Supplemental Figures


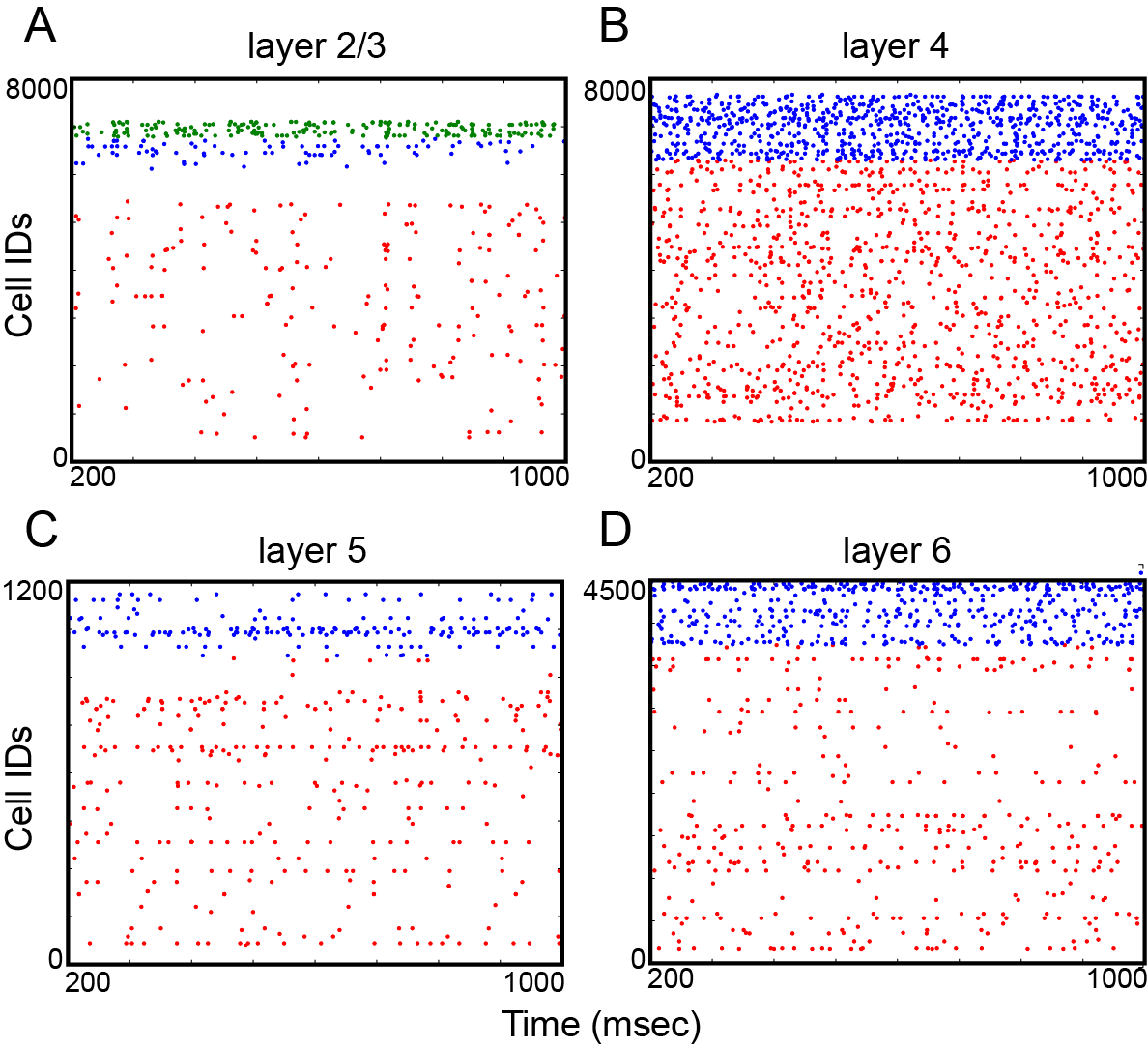


**Supplemental Figure 1: Spontaneous activity in all layers with parameters shown in Tables 1 and 2.** The action potentials in layer 2/3, 4, 5 and 6 are shown in **(A)**, **(B)**, **(C)** and **(D)**, respectively; 10 % of cells are recorded. The red dots represent action potentials of Pyr in layer 2/3 and excitatory cells in other layers. The blue dots represent action potentials of PV in layer 2/3 and inhibitory cells in all other layers. The green dots represent action potentials of VIP cells; in this experiment, SST cells are quiescent. Each row shows action potentials of individual cells, and cell IDs are nominal.


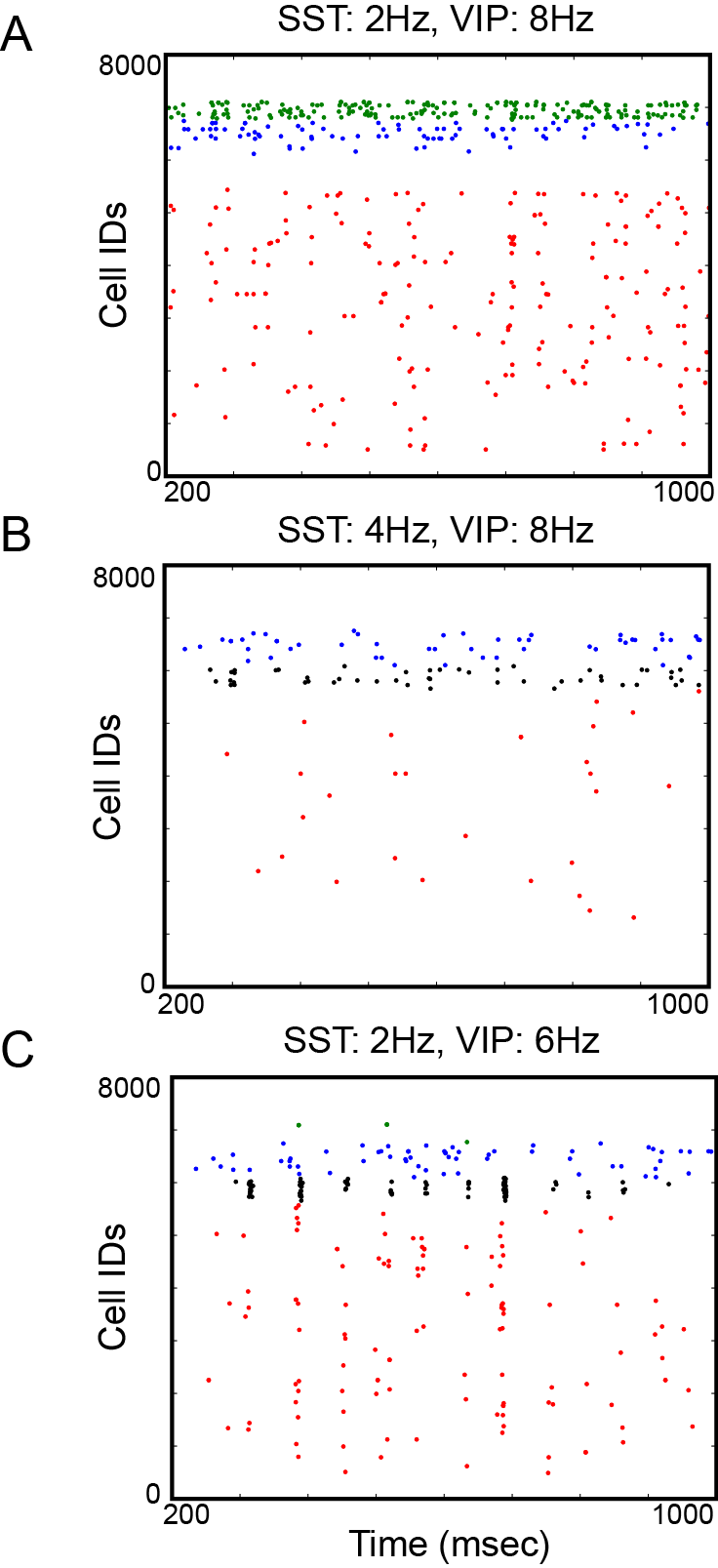


**Supplemental Figure 2: The dependency of layer 2/3 cell activity on background inputs**. The red, blue, green, and black dots represent Pyr, PV, VIP and SST cells’ action potentials, respectively. The chosen background inputs to VIP and SST cells (Table 2) are shown above each panel.


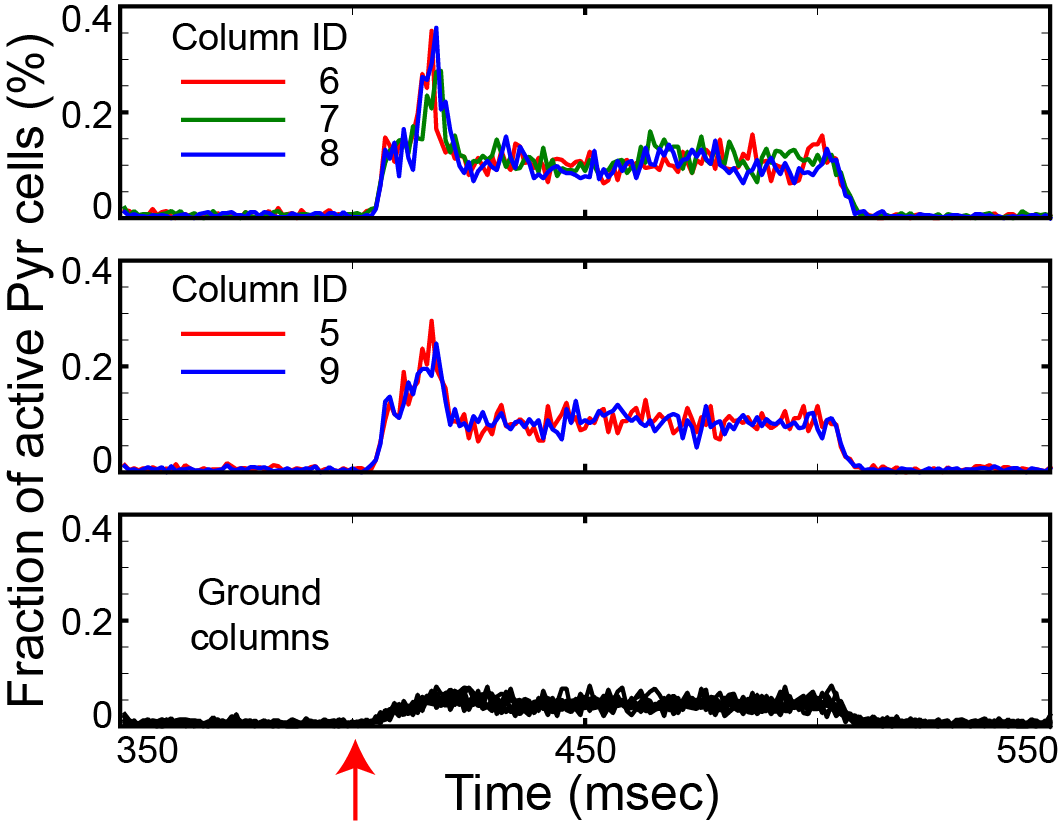


**Supplemental Figure 3: Time course of layer 2/3 Pyr cell activity in multiple columns.** The upper panel shows layer 2/3 Pyr cell activity in surface columns (6, 7 and 8). Pyr cell activity is averaged over 100 independent simulations. Similarly, the middle and lower panels show averaged layer 2/3 Pyr cell activity in edge and ground columns, respectively. All ground responses are shown in black. The red arrows represent the onset of thalamic inputs. The default parameters are provided in Tables 1 and 2. We counted the spikes using non-overlapping 4-msec bins; that is, 0.4% corresponds to 1 Hz. Layer 2/3 Pyr cells in an isolated column, shown in Fig. 3, fire at much higher rate due to the lack of lateral inhibition.


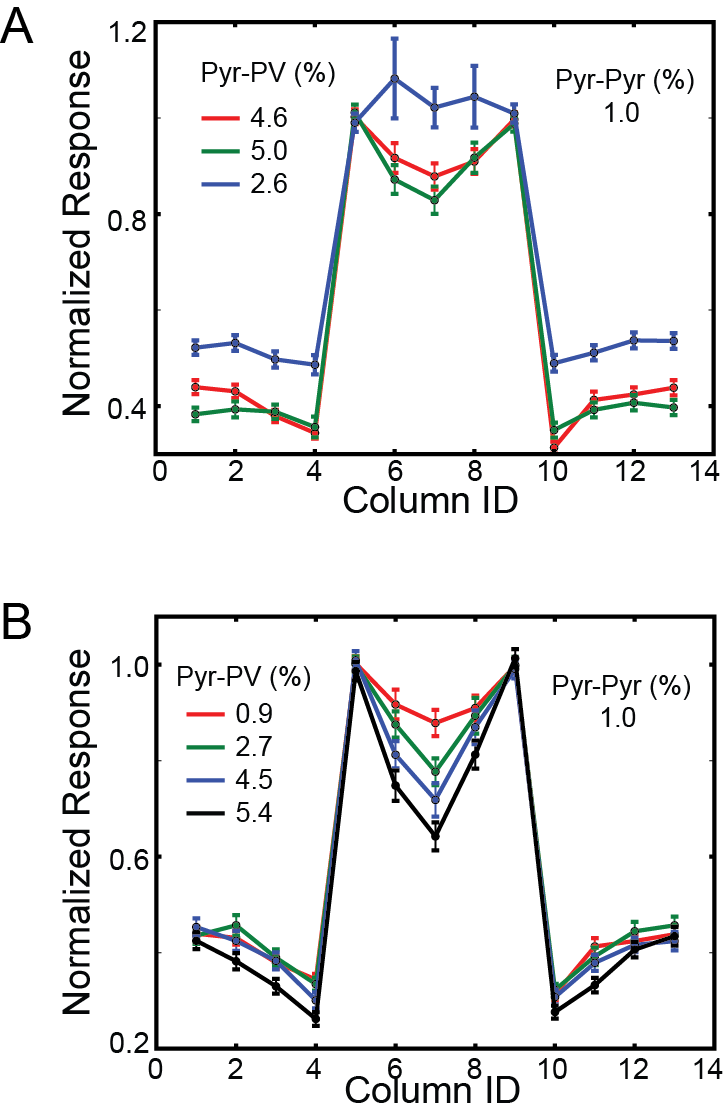


**Supplemental Figure 4: The effects of short-range inhibition on contextual responses**. Panels **(A)** and **(B)** show the same results as those in **Figure 3D** and **E** but with different connection probability for Pyr-Pyr cells. To compare the shapes of the response curve, we normalize the outputs using edge-responses for each connection probability to make all edge responses identical.


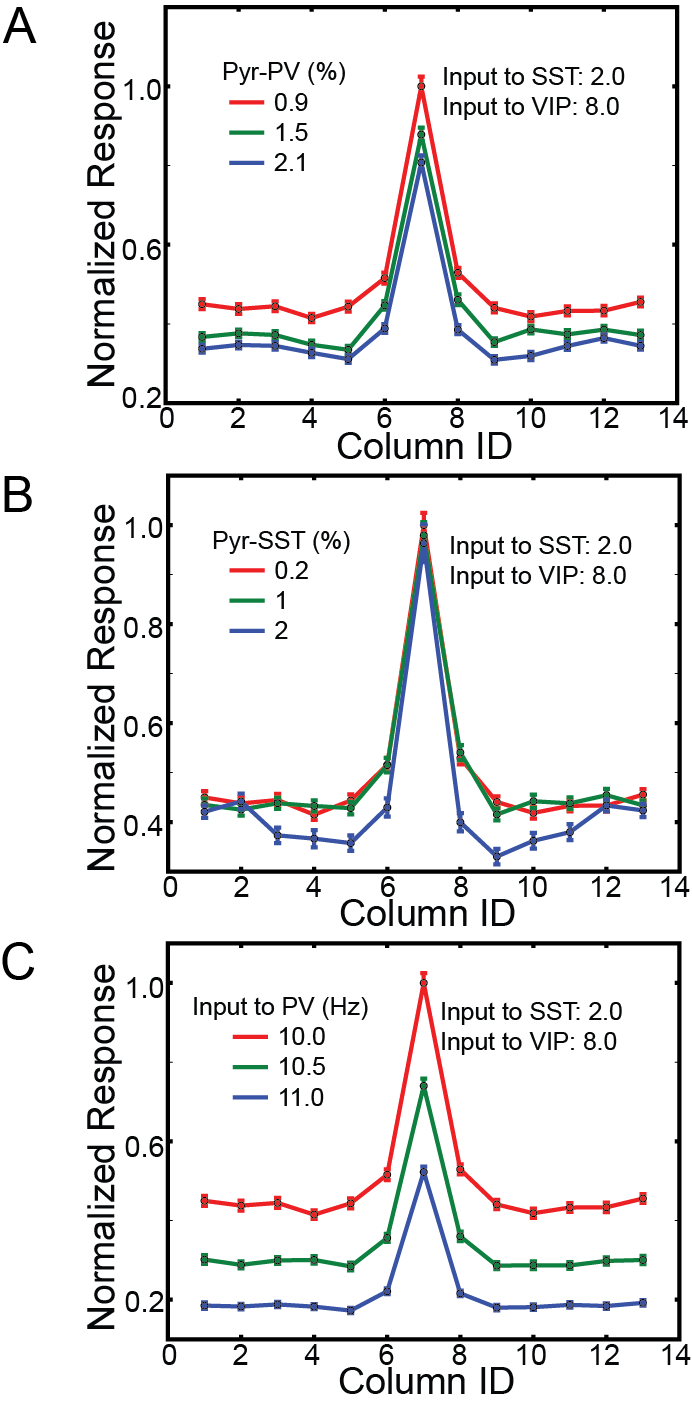


**Supplemental Figure 5: The effects of inhibition on shaping the tuning curve.** **(A, B)** Tuning curve can be modulated by short- and long-range inhibition across columns. **(C)** Subtractive effects are induced by enhanced inputs to SST cells.
